# Barriers and facilitators to implementing a Canadian shared-care ADHD program in pediatric settings in Shanghai: a consolidated framework for implementation research approach

**DOI:** 10.1186/s12913-024-10910-7

**Published:** 2024-05-02

**Authors:** Sayna Bahraini, Alexander R. Maisonneuve, Danping Wu, Minhui Huang, Mingyu Xu, Li Yang, Fei Li, André Samson, Feng Li, Philippe Robaey

**Affiliations:** 1 Children’s Hospital of Eastern Ontario Research Institute, Ottawa, Canada; 2 University of Ottawa, Ottawa, Canada; 3 Department of Developmental and Behavioral Pediatric and Child Primary Care, Brain and Behavioral Research Unit of Shanghai Institute for Pediatric Research, Shanghai, China; 4 Ministry of Education-Shanghai Key Laboratory for Children’s Environmental Health, Xinhua Hospital, Shanghai Jiao Tong University School of Medicine, Shanghai, China; 5 Ninghai Maternity and Child Health Care Hospital, Ning Bo, China; 6 Peking University Sixth Hospital (Institute of Mental Health), National Clinical Research Center for Mental Disorders, Beijing, China; 7 Key Laboratory of Mental Health, Ministry of Health (Peking University), Beijing, China

**Keywords:** ADHD, Shared care model, Barriers and facilitators, Consolidated framework for implementation research, Qualitative research

## Abstract

**Objectives:**

The vast majority of children with Attention-Deficit Hyperactivity Disorder (ADHD) do not have access to proper diagnosis and treatment in China. The goal of this project is to identify the challenges and facilitators in implementing a Canadian ADHD Shared Care Pathways program in pediatric settings in Shanghai region.

**Methods:**

Purposive semi-structured focus groups were conducted on a total of 13 healthcare practitioners from the Shanghai Xinuha, Ninghai and Chongming hospitals. Two independent researchers conducted a thematic analysis of the data with themes emerging based on the Consolidated Framework for Implementation Research (CFIR).

**Results:**

Notable barriers identified by participants included: (1) lack of knowledge in the management of ADHD, primarily among general practitioners; (2) lack of resources such as lack of staff, time, and medication for ADHD; (3) challenges in implementing an international multicentre intervention (such as communication difficulties between teams and integration of resources available in different hospitals); and (4) mental health stigma, difficulties in identifying ADHD patients, and logistical problems related to medication procurement rules put in place by provincial governments. Notable facilitators included: (1) the strong motivation of stakeholders and their confidence in their ability to learn and subsequently execute action plans to achieve the implementation goal; (2) the compatibility between the values and goals of the stakeholders and those of the program despite some cultural tension, a positive learning climate, strong tensions for change, and the high interest of organization leaders in engaging in the program (3) the perceived benefits of the program, such as standardization of the diagnostic and treatment process, and engaging primary care providers in ADHD management; and (4) the strong relationship between participating institutions and schools as well as provincial health initiatives available to support collaborative models of care. Mixed factors to implementation were also explored.

**Conclusions:**

Appropriate training of health care providers, cultural adaptation of the program, increase public awareness about ADHD to decrease stigma, as well as strong project management and guidelines that clearly describe the role and expectations of each team member appeared essential to successful implementation.

**Supplementary Information:**

The online version contains supplementary material available at 10.1186/s12913-024-10910-7.

## Introduction

Attention-Deficit Hyperactivity Disorder (ADHD) is one of the most common chronic childhood neurodevelopmental disorders, characterized by developmentally inappropriate inattention, hyperactivity and/or impulsiveness [1]. Widely accepted as a disorder in the Western world, despite ongoing controversies about its nature and treatment, particularly with medications, ADHD is increasingly being diagnosed within East Asia. ADHD has an estimated prevalence of 6.3% of children in China [2] and is associated with academic underachievement [3], emotional liability [4], and behavioural problems [5]. ADHD is highly heritable, with offspring having approximately a 34% chance of being diagnosed with ADHD when both parents are [6, 7]. It is a chronic disorder with 50% of those individuals diagnosed in childhood continuing to meet DSM-IV criteria for ADHD as adults [8]. In adults living with ADHD the condition often manifests as a sense of internal restlessness, deficits in higher-level executive functioning, and emotion control [9, 10]. This can often result in challenges in maintaining relationships, increased risk of substance abuse, and occupational difficulties [11–13]. North American, European, and Chinese ADHD treatment guidelines recommend a multimodal treatment approach which combines medication, education, and behavioural therapy [14–17]. Although we well know how to treat ADHD effectively, it remains difficult to treat well, because the outcome depends on a multi-modal, multi-professional, inter-agency approach, monitoring and adherence to treatment, and the management of other associated developmental disorders [18].

There are several challenges in the implementation of good-quality ADHD care in China. Such challenges can be viewed from both historical and cultural perspectives. China has undergone major economic reforms since the 1980s. Whereas previously medical services were accessed using rural community-led clinics and urban private hospitals, the Chinese medical system now comprises of different levels of hospitals, with varying levels of care spread around the country. Level 1 hospitals offer basic levels of care and are often located in rural communities. Level 2 hospitals offer increased care and make use of better equipment when compared to Level 1 hospitals, and are located in denser municipalities, districts, or provinces. Level 3 hospitals offer specialized care and are the best equipped health facilities within the country [19]. The level of education a physician has achieved in China dictates the type of hospital in which they may be employed. “Village doctors” hold a primary education level (1–3 years) and may work within rural communities. Physicians that hold secondary education (2–3 years) are permitted to work at Level 1 and Level 2 hospitals. Physicians with tertiary education (3 + years) may work in Level 2 and Level 3 hospitals. That being said, many hospitals within China require additional physician training in order to meet the requirements for employment [19]. The level of care that a patient receives is ultimately dependent on the treating physician’s level of education. These differences in education create problems with the diagnosis and treatment of children with ADHD within the country, resulting in delayed initiation and/or poor follow-up care for most patients. Primary care within Chinese urban settings is provided by General Practitioners (GPs) who are undertrained in general clinical psychiatry at the university level [20, 21]. As a result, these GPs often consider themselves not sufficiently competent to manage the diagnosis and treatment of children and adolescents with ADHD. Conversely, patients seeking mental health treatment often opt for specialist treatment within Level 3 hospitals without first seeking referrals from GPs due to a lack of trust in the treating abilities of GPs. This treatment seeking behaviour, coupled with an extreme shortage of specialists trained in ADHD management (approximately 500 specialists for 200 million children) creates an enormous burden in the mental health system within China. Patients are often faced with lengthy wait times for initial treatment and follow-up visits, must travel long distances to receive care (specialists are primarily located in dense urban areas) and in general, many do not receive the proper ADHD care that they require.

This shortage of specialists and inadequate training of general practitioners is not unique to China. In Canada, several systemic changes have been implemented to address these problems. For example, the Canadian Psychiatric Association and the College of Family Physicians of Canada have established strong collaborative bonds between family physicians and psychiatrists to promote timely evaluation and treatment for mental health conditions [22–25]. Patients with uncomplicated mental health problems are treated by GPs while consultation-liaisons by specialists meet the needs of the most complex patients [26]. Such a shared care approach can be generalized to other healthcare professionals such as pediatricians, nurses, therapists, and community resource managers to improve access to mental health care [27].

With this in mind the Canadian ADHD Shared Care Program is centered on CHEO (Ottawa) linked to local health services (especially family health teams) in the Champlain region stretching along the Ottawa and St. Lawrence Rivers. The program is based on four fundamental principles: the patient care pathway, shared care, stepped care and standardization of information. In the patient care pathway, evidence-based assessment and treatment takes place in four well-defined stages: (1) referral and pre-assessment screening (2), assessment through standardized evaluation questionnaires, a diagnostic interview, and a psychoeducational screening (3), initiation of treatment with a randomised placebo-controlled stimulant trial and/or a parenting program (4), a monitoring program with regular evaluation questionnaires. The program is mainly run by nurses, supported by secure web application for building and managing online surveys and databases, and producing standardized documentation. Stepped care ensures that treatment is delivered according to the patient’s needs. The most effective and least resource-intensive treatment is provided by primary care providers, while more intensive and specialized services are used more frequently as the severity of the condition increases. Shared care relies on effective collaboration and communication, based on defined criteria, between primary and specialist care providers, and between care providers, young people and their families. Finally, standardized communication ensures the efficient and reliable exchange of information within and between care teams and the family/patient. This enables the patient to move from one stage to another and from one level of care to another in the most cost-effective and timely manner.

In China, the vast majority of children with ADHD do not have access to timely diagnosis and treatment in China. A shared-care approach could address several difficulties in the management of children with ADHD in China by addressing the scarcity of specialists, the complexity of a systematic approach to ADHD, and the lack of training of PCPs. In line with Chinese guidelines for diagnosis and treatment [17], the program also provides multimodal treatment combining educational, behavioral, and pharmacological approaches. This program promotes shared responsibilities between PCPs and specialists within a well-defined care pathway. Within this shared-care perspective, it is essential to define which specialists are best able to successfully collaborate with PCPs and in what setting. In China, despite progress over the last decades, there is an extreme dearth of child psychiatrists overall [28, 29]. In a previous study, we examined the barriers and factors to implementing an ADHD treatment program in the Beijing psychiatric environment [30], where a level 3 psychiatric hospital (Sixth Hospital of Peking University) had entered into a partnership with Haidian Mental Health Hospital and associated community hospitals to improve the management of mental health services in lower-level hospitals. However, to address this shortage of specialist, pediatricians and some GPs also are a resource, as they are encouraged to train in the early diagnosis and basic treatment of the most common mental health disorders in childhood. Moreover, Developmental Behavioral Pediatrics is recognized as a pediatric subspecialty in China. Developmental behavioral pediatricians have specialized training and expertise in the assessment and care of children with a broad range of developmental, learning, and behavioral difficulties, including ADHD, and may have an easier connection with GPs and pediatricians.

The starting point for the project was the involvement of behavioral development pediatricians in ADHD care in a level 3 hospital, and the need to develop collaboration and training programs with other health professionals in level 2 and 1 hospitals. The joint Ottawa-Shanghai medical school was already co-established by the Shanghai Jiao Tong University School of Medicine and the University of Ottawa Faculty of Medicine. In this context, researchers from both universities seized the opportunity of the joint health research initiative of the Canadian Institutes of Health Research (CIHR) and the National Natural Science Foundation of China (NSFC), under the auspices of the fourth joint program of the Global Alliance for Chronic Diseases (GACD), which focused implementation research on all mental disorders beginning in childhood, adolescence or adulthood. The developmental behavioral pediatrics department at Xinhua Hospital Level 3 (affiliated to the Shanghai Jiao Tong University Medical School) was already collaborating with developmental behavioral pediatrics departments in two Level 2 hospitals: the Xinhua Hospital Chongming Branch (in the municipality of Shanghai, but a two-hour drive from downtown), and the Ninghai Maternity and Child Care Center (in Zhejiang province, 280 km from Shanghai), themselves associated with regional community hospitals (levels 1 and 2). Therefore, in this study we aimed to identify the challenges and facilitators of implementing this program between level 2 and 3 developmental behavioral pediatrics services.

## Methods

A purposive sampling method was used to select participants who would provide valuable and informative perspectives on the implementation of a Shared Care Pathways program in Shanghai. The Investigator local to Xinhua and Chongming Hospitals (F.L.) and the Investor local to Ninghai (M.H.) used their understanding of local contexts to select individuals who would be most likely to identify the barriers and facilitators involved in implementing this project. Three semi-structured focus groups (FG) were conducted with a total of 13 healthcare practitioners from the Shanghai Xinhua hospital (FG#1: *n* = 2 GPs & 3 specialists), Chongming hospital (FG #2: *n* = 2 GPs & 1 specialist), and Ninghai hospital (FG#3: *n* = 3 GPs & 2 nurses): see Table 1 for characteristics of the research participants.

**Table 1:** Characteristics of the research participants

Role	Extra training on ADHD management	Work experience in years (In general/the current care setting)	Usual Work Setting	FG	Qualification
GP	Yes	21	Secondary Hospitals/Dong Yang Maternal and Children Hospital	1	Bachelor’s Degree/Chief Physician
GP	No	28	Secondary Hospitals/Chongming Branch of Xinhua Hospital, School of Medicine, Shanghai Jiao Tong University	1	Bachelor’s Degree/Attending Physician
Specialist	Yes	16	Tertiary Hospitals/Xinhua Hospital, School of Medicine, Shanghai Jiao Tong University	1	Ph.D. Degree/Chief Physician
Specialist	Yes	22	Tertiary Hospitals/Xinhua Hospital, School of Medicine, Shanghai Jiao Tong University	1	Ph.D. Degree /Associate Director Physician
Specialist	Yes	15	Tertiary Hospitals/Xinhua Hospital, School of Medicine, Shanghai Jiao Tong University	1	Ph.D. Degree/ Associate Director Physician
GP	No	40	Secondary Hospitals/Chongming Branch of Xinhua Hospital, School of Medicine, Shanghai Jiao Tong University	2	Bachelor’s Degree/Chief Physician
GP	No	28	Secondary Hospitals/Chongming Branch of Xinhua Hospital, School of Medicine, Shanghai Jiao Tong University	2	Bachelor’s Degree/Attending Physician
Specialist	Yes	16	Tertiary Hospitals/Xinhua Hospital, School of Medicine, Shanghai Jiao Tong University	2	Ph.D. Degree/Chief Physician
GP	No	21	Ninghai Maternity and Child Health Care Hospital	3	Bachelor’s Degree/Chief Physician
GP	No	21	Ninghai Maternity and Child Health Care Hospital	3	Bachelor’s Degree/Chief Physician
GP	No	23	Ninghai Maternity and Child Health Care Hospital	3	Bachelor’s Degree/Associate Physician
Nurse	No	12	Ninghai Maternity and Child Health Care Hospital	3	Bachelor Degree/Nurse
Nurse	No	3	Dong Yang Maternal and Children Hospital	3	Bachelor Degree/Therapist

### Data collection

In this study, qualitative data was collected through semi-structured focus group interviews. A semi-structured interview allows interviewers to explore themes that are particularly important to the participants, while allowing for a structured approach. Using nine interview questions and specific probes, the Canadian researchers collected information about the current state of ADHD care along with facilitators and barriers that may support or hinder the implementation of Shared Care Pathway program locally (see supplemental material for questions). The interview questions were translated into Chinese using the adapted WHO guidelines for cross-cultural translation (forward translation, initial cross check, backward translation, and final cross-check). Chinese researchers facilitated the focus groups in face-to-face or online sessions. We translated the transcripts into English and conducted the data analysis.

### Data analysis

#### The Consolidated Framework for Implementation Research (CFIR)

The CFIR provides a variety of constructs arranged across five domains that can be used to systematically assess potential barriers and facilitators in the process of implementing a new intervention. This framework further allows for the development of context-specific logic models and generalizable theories [31]. Such a framework has been used in hospitals, pharmacy, dentistry, primary care, and behavioural health agencies [32, 33]. This framework has also been used in conjunction with the identification of barriers and facilitators in healthcare implementations in China [30]. A complete list of CFIR constructs and domains used within the realm of this research can be found in Appendix A.

#### Qualitative approach

A quasi-deductive approach comprising of both inductive and deductive approaches was used for data analysis [34]. Such an approach has seen success in identifying barriers and facilitators in qualitative health research (see [35, 36]). Using CFIR constructs and definitions as a guide, transcripts were first deductively coded without inferences made from the data. Following this, a thematic inductive approach was applied to the codes previously generated, identifying sub themes within the CFIR domains. An inductive thematic analysis [37] is an appropriate and powerful method to use when seeking to understand a set of experiences, thoughts, or behaviors across a data set [38]. This allows researchers to search for themes of broader significance that was provided from the deductive coding, producing a rich description of the analysis. Data analysis was completed using the NVivo qualitative software package (version 12).

#### Data integrity

Several measures were utilized by the researchers to improve credibility and trustworthiness of the analysis including continuous comparative analysis and immersion in the topic of study [39]. Dependability was adhered to through the consistent execution of study procedures. Furthermore, transferability was increased through providing information on the context in which the research was carried out, research participants, and methods [39]. To enhance confirmability, decisions made during the research process and emergence of the findings were reviewed in joint meetings with Canadian and Chinese researchers. We conducted member checking to increase credibility. As our objective was to adapt and implement a Canadian model of care in China, which have two very different cultures and health care organizations, we used a multi-coder approach to ensure methodological rigor. Two coders (members of the Canadian team) independently analyzed the data and came to a consensus on the results of the analysis. An additional researcher from the Chinese team (trained by the Canadian team) independently coded the transcripts, and all three researchers reached an agreement on the barriers and facilitators they identified. Conflicts regarding coding and themes were resolved in joint meetings between Canadian and Chinese researchers.

## Results

Based on the CFIR, constructs pertaining to the following domains were identified: *Characteristics of the Individual*, *Inner Settings*, *Intervention Characteristics*, and *Outer Settings.* Table 1 (provided in the Appendix) summarizes the identified facilitators, barriers, and mixed factors related to each construct.

### Characteristics of individuals

#### Knowledge and beliefs

The majority of participants pointed to lack of knowledge of GPs regarding ADHD, and their unfamiliarity with accepted procedures and principles of ADHD management which resulted in the immediate referral of potential cases to specialists: *“I think GPs are not familiar with this disorder [referring to ADHD] and will not accept any cases. They will tell you to go to XX hospital [a third level hospital] instead of thinking how they can help [manage the case]”*. Relatedly, the majority of participants believed that they need training as well as support from specialist in other fields for complex ADHD cases: *“…However, some patients may have other comorbidities. In those cases, we need to work with paediatric psychologists… for consultation, discussion, or even referral. We need to encounter more cases to be able to handle complicated situations*”.

### Self-efficacy

Participants showed moderate to high levels of confidence in their capability to implement the project. However, participants indicated that they required support from various team members, as well as their administration in order to do so: “*We are confident since we are at this level. However, we need help from the upper level of administration in a lot of areas such as technical support. In other words, we need appropriate coordination*”. Participants expressed the desire for additional training on non-medical treatment approaches and the details of the Shared Care model.

### Other personal attributes

Participants expressed a genuine desire to implement the project and help their patients: *“Well, I think, ADHD patients show various problems in behaviour and study. Any improvement they show will greatly encourage you. That is where my motivation comes from”.* Other sources of motivation for participants in implementing the program were: enhancing their level of diagnostic and treatment skills, upgrading their academic level by being involved in the project, standardizing the management of care, and eventually improving the economic benefits to their hospitals.

Participants placed a high value in enhancing knowledge in their field by allocating time to research and sharing new knowledge with other team members: “*My team members’ eagerness and devotion to science has made me more confident. They are using their spare time to do this project…motivation is essential*”. Another participant mentioned: “*Even though we are so used to what we have been doing in the out-patient clinics, every time we learned a new concept, we talked about it in our department”.* In terms of learning style, participants mentioned that their main preferred method of learning was through role-playing, followed by online training sessions, and educational videos.

### Inner setting

#### Implementation climate

Compatibility: Participants felt that the goals of the project were generally congruent with their own values. They believed that the project would significantly improve treatment of ADHD patients, which is consistent with the goals and values of health care providers: “… *whatever the trainers talked about was highly practical and compatible with our work. Some of them [the training content] are already in our practice. Some of them are things we need to pay attention to*”. In addition, a participant who identified themselves as a leader in their health care setting was receptive and supportive to the project, citing the need to learn and develop: “*I am sure that the whole community in our Chongming area lack knowledge about the disorder because we got little training. I will support this project. It is a great opportunity for us to learn and get trained*…”. Although participants believed that there is a great compatibility between their own values and the project’s goal, they noted that the philosophy underling ADHD treatment is different between Canada and China. Specifically, participants mentioned that the current care approach in China emphasizes supporting children’s abilities to meet strict expectations. Participants perceived the Canadian approach as being comparatively more focused on trying to understand the limitations that are driving children’s behaviour and adjusting accordingly. However, the majority of participants expressed that a more empathetic approach could be beneficial in mental health treatment: “*I think we need to make some changes in our philosophy. Different educational concepts have led to different values and beliefs. We will become more open after learning. I think we need to put ourselves in the child’s shoes and try harder to understand the child. That will be more helpful to the child*…”.

Organizational Incentives: Although salary increases and bonuses were mentioned, participants placed more of an emphasis on increasing respect through obtaining certification for completing ADHD training sessions through the project, as well as participating in scientific research and writing articles: “*People from the Psychology Department always clamour that you cannot run such programs because you don’t have the license… can you give us some certificates for promotion, to prove that we can run its program after training… Yes, give us some certificates so that we can put them on the wall.”* In addition, participants mentioned the training in new research methods as an additional incentive: *“Personally, I am quite interested in qualitative research… but I have no idea where to start. It would be a great experience if I can learn something like that in this project”.* Other incentives included allocating more trained staff (regular and skilled employees) to help with ADHD assessment and screening, and receiving financial promotions.

Learning Climate: Regarding this sub-construct, participants who were recognized as leaders within their organization spoke of their own need to learn and collaborate with colleagues: *“Even though we are psychiatrists… even though you recognized their conditions [ADHD children] and planned to treat them, you will be frustrated at the outcome… I need further training in what to do to help them”.* From a more logistical standpoint, participants discussed different training opportunities that promoted a positive learning environment for them. One of the leaders mentioned: *“…We were the first group to get the training. We have also got online training from Professor [Name]. Through this training, we have enhanced our level of professional skills and we have a better idea about care management in our county level hospital”.* Although leaders demonstrated their desire to learn from other team members, there was an implication that participants were more willing to learn from colleagues who have equal or more senior positions than themselves, and not junior colleagues.

Tension for Change: In our study, participants showed a strong urge for change and subsequent desire to implement this project as it contributes to parents, doctors, and teachers’ knowledge in ADHD recognition and management: *“As doctors in the communities, we have to enhance our ability to make correct diagnoses. We have to be able to tell what is mild, what is serious, what is complicated or with comorbidities”.*

Given the patient age ranges that Chongming community hospitals usually serve (which is younger than the age that most ADHD diagnoses are made) this project would also address issues related to ADHD diagnoses in older children but locally. As one participant pointed out: *“Currently township hospitals are dealing with the population from 0 to 6-year-old and that is also related to their level of skills.”* Instead of being treated locally, older patients must travel to Shanghai every two weeks to be treated by institutions that treat patients over the age of seven, and subsequently patients who eventually receive an ADHD diagnosis do not need to travel. Ultimately, participants felt that the implementation of a Shared Care approach to ADHD management would enable them to treat their patients locally, rather than referring them elsewhere, which could lead to a *“reduction of burden to the economy”*.

#### Readiness for implementation

Leadership Engagement: Regarding this sub-construct, many of the participants (who are considered leaders in their respective occupational settings) indicated their interest in engaging in the project to train community doctors: *“We are now talking about forming collaborations to help each other. Doctors in the communities are not skillful enough to deal with the disorder, right? I can teach and train them with the practical ways*”. Similarly, one of the leaders in Xinhua Hospital [Third Level Hospital] indicated their team’s willingness to coordinate and manage the interdisciplinary team in other designated hospitals: “*We will coordinate the interdisciplinary team to provide excellent services. I talked to X [name of one of the potential leaders in Shanghai] yesterday. I said we are not the focus in this project, but the two regions we serve are [pointing to Chongming and Ninghai]*”.

Available Resources: To begin, participants indicated that the level of healthcare provided in the communities of Chongming and Ninghai are usually very basic and is often limited to growth monitoring and immunization. One participant mentioned that: “*Although they also have primary childcare in the community, the service is relatively simple… it is only limited to height and weight measurements”.* Another participant mentioned that: “My job is basically doing the assessments… The rest of my job is communicating with the doctors.”

Participants spoke about the insufficient resources needed to treat paediatric ADHD, particularly with regards to qualified personnel: “*No pediatricians are specialized for ADHD in this hospital [referring to a secondary level hospital]. No one is specialized in overall developmental behavior”.* In addition to lack of specialists, participants indicated a lack of “regular and qualified staff” to assist with assessment. Specifically, more staff with appropriate training could conduct routine assessments and therefore reduce the workload of specialists. For example, one participant indicated that: “*Only I and [Name of the doctor] are doing the assessments. Nobody else is qualified, has a certificate.”* It was further mentioned that: *“Because we use Wechsler Intelligence Scale and SNAP-IV for screening, it takes long time to finish one assessment, and the community nurses don’t know how to do it. They need training, and the training takes time too.”* Because of this lack of trained GPs and nurses, many of the hospitals surrounding Shanghai shift the focus to ADHD management: *“Basically, this is the reality. Personally, I think it is somewhat difficult for us to reach out to the communities to do assessments. It would be better if we focus on promotion and education. This way is better for them too.”* For the project to be successful, participants indicated that it would be necessary to determine the level of expertise of GPs regarding ADHD and find new ways to increase their interest in the topic.

The heavy workload and lack of time for pediatric specialists in third-level hospitals were also reported as challenges for project implementation and generalist training. One of the specialists said, “*Honestly, I feel almost exhausted from the clinical work. I have to see over 30 new patients in one morning…*”.

A lack of standardized procedures to ADHD diagnosis and treatment was identified by participants as another barrier. Participants repeatedly spoke of a piecemeal approach to ADHD that often varied from hospital to hospital and from physician to physician: *“Because this specialty, ADHD assessment, belonged to the neurology or psychology department in the past. It was not in the primary child health care system until recently. In addition, the number of ADHD patients is relatively small. Doctors are actively developing [their own practices]… The care management for those children is not systematic, and the number of those children going to the hospital pediatric department is also relatively small.”*

Participants expressed the need for clear guidelines on ADHD treatment throughout China*“… Actually, we have made some referrals. However, without a guideline, all the decisions were made based on an individual doctor’s judgment. Therefore, we need to make the procedures standardized*”. To mitigate these barriers somewhat, participants felt that the materials that would be available through the implementation of our proposed program would support standardization: “*Given the resources available now, including the educational models* [referring to the program], *I think it is quite easy for us to achieve that goal* [standardizing the procedures]”.

Finally, participants identified the unavailability of ADHD medication at primary and secondary level hospitals as a barrier: “*At the level of county hospitals… not all the hospitals can purchase the medications and keep them in inventory*”. Similarly, another participant mentioned, “*If the patient needs pharmaceutical treatment he goes to (a third level) hospital to get the medication*…” For hospitals that do have the capacity to obtain ADHD medication the logistical barriers to maintaining an adequate supply are substantial: “*If they want to use this medication, they can apply for the interim license… you can submit a report when you want to use this medication…”* These provisional licenses are only valid for limited periods and quantities: “*The application process lasts for one month. But how long they can use one depends on how much medication they will use. For example, they can’t stock up a lot of medications in their hospital in one application”.*

### Intervention characteristics

#### Complexity

The participants felt that collaborating and communicating between the teams of different countries, and different hospitals, integrating the resources available in the different hospitals, and meeting the needs of these different hospitals was a real challenge in finalizing this project within five years: *“However, how to better implement and carry on for 5 years remains a challenge…This is an international project. We are going to transplant a new system from a foreign country. Cooperation and communication will be a huge challenge.”*

#### Intervention source

On a related note, our research participants acknowledged that the intervention itself was international in nature and therefore “*foreign*” and may need some adjustment to become appropriate to the Chinese context. However, some participants had confidence in a project developed in Canada: “*The program has been developed in Canada…. I am confident about that system”.* Despite the foreign source of the intervention, all participants indicated that they were willing to engage in its implementation: “*I like that this is an international project. Through this project we can learn from the foreign advanced experience and apply it to our practice*.” When discussing the benefits of the training that was provided as part of the implementation of this project, participants said: “*From what we learned yesterday and the other days [from researchers in Canada], not only did I learn more about the knowledge network in psychiatry, but also about project management.”*

#### Relative advantage

Participants indicated that our project had the potential to help not only with the standardization of ADHD treatment, but also with the administrative management of working with these patients: *“…this project is very good because there is already something similar in place [in Ninghai], which is a good foundation. The new project [current project] can optimize the flow of the procedures which are already working, making them more efficient.”* In addition, participants felt that the involvement of GPs was a strength of the project: “*The pediatric department is responsible for training the general physicians. So, they have the advantage. They can take that opportunity to further train their GPs”*.

### Outer setting

#### Cosmopolitanism

Several participants from secondary/tertiary hospitals reported well-established working relationships with other hospitals in the region, and with university teaching hospitals in Shanghai. Participants also reported strong working relations with schools in the area: “*Generally have very good relationships with elementary schools”* which could help promote the program. Expanding on this, another participant indicated that health promotion through schools was effective *“We have over 100 elementary schools and high schools… it may also be done [health promotion] through the parents in the schools”.* Leaders from local hospitals also often used their personal connections to promote knowledge of ADHD in schools: *“.… Currently our Director [name] is using his personal network to do promotions. For example, some of his classmates/friends are working in schools, and they invited him to the schools [for educating teachers about ADHD]”.* Therefore, building a formal connection with schools in Ninghai has been considered as an area that needs to be improved for implementation of the project.

In addition, participants indicated that they could invite senior physicians from teaching hospitals to train junior physicians: *“In terms of knowledge network,… another resource available is the teaching hospitals. Teachers in hospitals affiliated to the university will come for this project”.* It was indicated that some hospitals could share best practices between their respective institutions, allowing for optimal training levels and treatment approaches: “*Shared information. There is a lot of information about how to enhance attention [in kids with ADHD], I know there are materials specific on this subject. I wonder if we can share related information from big hospitals.”* Finally, participants from Ninghai Hospital indicated that they had an excellent working relationship with other community hospitals: “*We have a very close relationship with the 17 township hospitals around us… Whenever we have new or special cases we will communicate within the group. It is very convenient”.*

#### Patient needs and resources

Most participants indicated that there is a significant lack of public awareness of ADHD in Chongming and Ninghai: “*Education to raise people’s awareness is needed, and relatively we are falling behind in this area.… I think generally parents, doctors, and teachers are not updated, and that is where we need to make some improvement*”. This lack of awareness has been mentioned as one of the main obstacles in identifying and treating patients with ADHD in Chongming and Ninghai: *“There are approximately 2000 children with ADHD in Chongming* [estimation based on the prevalence of ADHD in paediatric populations] *…, but it is challenging to identify them all… where are these 2000 patients?”* Participants indicated that a significant number of social resources, spanning all levels of society, would be needed to implement the program. Furthermore, even among those who know about ADHD, many do not know that treatments exist and are available: *“Despite the large-scale publicity campaign, there are still a lot of people that don’t know that ADHD is treatable. They don’t know treatment is available in their community hospitals either.”*

This lack of awareness was attributed to limitations in Chinese primary health care for children: *“This hospital is the only hospital with a primary childcare department on this island [Chongming], and the primary childcare department focuses mainly on the kids under three years old. So, we can see that people on this island lack education [on ADHD].”* Socio-economic factors were also shown as a limiting factor: *“The differences here compared with Shanghai are that the economy here is backward and the parents here are less educated. The parents’ consciousness is still different from that of Shanghai people… the educational level of the teachers is pretty good in Shanghai and Chongming island is relatively behind. Its social economic status is pretty low.”*

Finally, participants reported that their patients were often wary of the stigma associated with mental health in China, including for pediatric ADHD. For example, participants reported that parents often chose to go to pediatricians in a primary care setting rather than to a mental health specialist: “*if the children have the ADHD problem, they are willing to go to the pediatrician, and they are more willing to go to the primary childcare department, because the primary childcare department could see all kinds of patients. But, if the kids go to the mental health hospital, they have to be documented.”* From the same perspective, people living in Chongming would prefer to go to Shanghai to reduce the risk of their child’s ADHD diagnosis becoming known in the community: “*Even if they feel something wrong, they will rather not say it;… rather not come here for treatments; but they might go to see doctors in Shanghai city secretly. Because this is a relatively small place, it will be spreading among people soon”.*

#### External policies

Policies at various levels of government to increase general practitioner training within the pediatric departments of Chongming hospitals was identified as a facilitating factor: “*The Shanghai government has given the order that GPs on this island have to get some training in the pediatric department*.” Related to this, and with the growing demand of medical services within Chongming, the municipal government of Shanghai took additional measures: *“as we are far away from downtown Shanghai, and at a special geographical location, people’s demands for both medical services and the general services provided by our hospital are still growing. Therefore, in 2009, Shanghai municipal government authorized Shanghai Jiao Tong University and Xinhua Hospital to take full charge of the management.”*

## Discussion

The purpose of the current study was to determine barriers and facilitators of implementing a Canadian ADHD Shared Care Pathway program in pediatric settings in Shanghai. We collected the data from healthcare providers who work at the hospitals where the project will be implemented in the future. Using the CFIR, a variety of domain and constructs were identified that support or hindered the implementation of this project. Regarding the domain of characteristics of the individual, knowledge and believe about the intervention, self-efficacy and other personal attributes were explored by participants. Related to Inner Setting, the implementation climate was spoken of at length. Specific to Readiness for Implementation, one of the most significant talking points was leadership engagement and a lack of available resources. Specific to intervention characteristics, complexity, the source of the intervention, and the relative advantages were explored. Rounding off the domains covered by the CFIR, the Outer Setting was highlighted insofar as it pertained to cosmopolitanism, patients’ need and resources, and external policies. The mental health stigma and lack of public awareness about ADHD were spoken of at length by focus group participants as the main barriers in this domain. Key factors under each domain will be further discussed.

### Characteristics of individual

GPs’ limited knowledge about ADHD management in Shanghai has been identified as one of the key barriers in implementation of the ADHD Shared Care Pathway program. This gap may be attributed to GPs’ insufficient education about ADHD and developmental behavioral pediatrics in universities, lack of experience with pediatric population, and limitations in prescribing medications. Similarly, in a systematic review conducted by Tatlow-Golden and colleagues [40] lack of ADHD knowledge has also been reported by GPs in Australia, UK, Canada, and Iran. Results of this study revealed that almost all participating GPs in the aforementioned countries had low confidence in ADHD diagnostic ability and believed the overview of specialists was required for ADHD management. In line with the result of the current study, GPs cited lack of training and uncertainty regarding ADHD principles as main reasons for their limited knowledge. Despite and perhaps because of the participating physicians’ inadequate knowledge of ADHD, our results revealed their strong desire to learn. Developing a training program that is designed based on the different physician’s level of knowledge can improve their understanding and practice toward ADHD management [41]. As Chinese physicians’ heavy workload can be a significant barrier for attending in training programs, a combination of on-line and web-based ADHD training programs can offer easily accessible training at the time and place of physicians’ convenience.

### Inner setting

Compatibility of the Shared Care Pathway program with stakeholders’ values and beliefs has been identified as a great facilitator for implementation of this project in Shanghai. According to the CFIR [31], the more knowledge users perceive compatibility between the innovation and their own values has a strong impact on likelihood of their acceptance of the innovation. This factor has been considered as an important predictor of a successful implementation. However, clear cultural differences have also emerged, particularly in non-pharmacological approaches to ADHD. In the West, and specifically in Canada [42], individualized educational plans are offered to children with neurodevelopmental disorders (“exceptionalities”), with curriculum adaptations or different expectations to help students acquire skills that are not included in the curriculum, or that are consistent with the child’s actual learning level. The concept of individualization acknowledges the child’s limitations, for example in attention or motor control, and considers behavioral problems as a response to the mismatch between the child’s abilities and adult expectations. From this perspective, the role of the adult (parent or teacher) is to adapt their expectations so that the child can be successful again. In the Chinese society, shaped by Confucianism despite Western influences, the role of parents is to transmit to their children a sense of responsibility for academic success, obedience and maintenance of harmony in the home and classroom [43, 44]. Problems of inattention, hyperactivity or misbehavior are primarily perceived as a failure on the part of parents or teachers to transmit this sense of responsibility and self-control. The more adults blame themselves for their children’s failings, the harder it is for them to match their expectations to their children’s abilities, because that’s precisely what they blame themselves for: failing to instill these expectations in their children. Conversely, in the West, the main resistance to accepting individualized education plans often comes from the children themselves, who resent the fact that expectations are different for them as compared to their peers, because they want to be like everyone else. In this perspective, pharmacological approaches did not elicit the same feedback from participants, since the blame lies on a cerebral dysfunction in the child, which the treatment aims to correct to enable the child to meet expectations. These cultural differences are reflected in the current lack of accommodation for children with ADHD, particularly in the school environment. The implementation of a shared model of care therefore has to cope with significant cultural differences, especially around the issue of accommodation. Encouragingly, however, participants recognized the benefits of taking the child’s perspective (“putting yourself in the child’s shoes”). The enthusiasm of participants to learn and apply Western approaches to the diagnosis and treatment of ADHD is an important facilitator of successful implementation. Nevertheless, the research team became acutely aware of the need to culturally adapt the program so that it would be acceptable in the Chinese context. In the next phase of the project, we decided to use a cultural adaptation framework for all the different components of the Shared Care Pathway program. This cultural adaptation framework will permeate all training activities. For example, we started to culturally adapt an ADHD diagnostic interview during training. At each step of the training program, feedback is obtained on cultural adaptation, and provided to a steering committee formed of Chinese knowledge-users that ultimately decide which to include.

Adaptation of the program will also address some of the other identified barriers such as lack of resources due to modification of the program to consider hospitals’ supplies and capacities. Other identified facilitators in the inner setting, such as positive learning climate and strong leadership engagement in participating hospitals are also considered as factors that will increase success of the implementation.

### Intervention characteristics

The key barrier identified under this domain was complexity of conducting an international implementation project. There is a negative relationship between knowledge users’ perception of complexity of the interventions and success of implementation [31, 45, 46]. Therefore, determining complexity of the project and applying strategies that contribute to successful implementations is of a great importance. An international project is always complex to organize, and participants placed particular emphasis on the need for communication and cooperation. The main challenges are geographical distance, which limits opportunities for face-to-face contact; language barriers (difficulties with English as a working language, lack of knowledge of Mandarin, need for simultaneous translation, captioning of audiovisual documents), particularly outside university centers, where there are fewer foreign-trained professionals fluent in English; and time differences, which limit the time and duration of videoconferences. Added to these linguistic difficulties is the perception of the program as foreign, and the need to adapt it so that users can make it their own. Suggested by CFIR [31], having simple, clear, and detailed implementation plans, schedules, and task assignment are some of the strategies that facilitate the process of implementing a complicated project. In the present project, limiting its size to a collaboration between a Level 3 center and two regional Level 2 centers, a clear definition of shared care and the care pathway are key success factors. If the international aspect of the project was perceived as a difficulty, it was also felt to be a facilitator, because the stakeholders had confidence in a program developed in Canada and were willing to learn from a foreign experience and apply it in their own practice.

### Outer setting

Stigma and lack of public awareness of ADHD were also identified as key challenges for the project’s implementation in Shanghai. Stigma is one of the factors limiting access to and use of mental health services [47]. The stigma towards mental health problems can be felt all over the world. A recent systematic review of attitudes towards ADHD in community samples from Australia, Sweden, Germany, Finland, Korea, Indonesia and the USA found that attitudes are generally negative [48]. ADHD is considered to be overdiagnosed, the acceptability of ADHD drug treatment is questioned, and people with ADHD are thought to be more likely to exhibit inappropriate behaviours, and best kept at a distance.

In China, the disruptive behaviors often associated with ADHD clash with the expectation of harmony in the classroom, necessary for the concentration of other students. Disruptive children are often rejected and sometimes excluded from the school environment. The stigma and rejection are all the stronger when ADHD is not recognized or treated, and when adaptation efforts are lacking. The project to implement the Shared Care Pathway model in the pediatric setting may help to reduce stigma. China is one of the few countries where Developmental Behavioral Pediatrics is recognized as a subspecialty. The Academy Section on Developmental and Behavioral Pediatrics in China was only founded in October 2011. Parents who feel too embarrassed to consult a mental health center for fear of the stigma attached may more comfortably seek care in a pediatric service.

The hospitals in our study have already established close links with schools and are running neurodevelopmental health promotion workshops on ADHD with parents and teachers in the school setting. Improving the level of mental health literacy is supposed to reduce stigma and discrimination against people with mental health problems [49]. In the West, non-professionals, particularly teachers and parents, generally have a poor understanding of ADHD, and tend to endorse biological, rather than psychosocial, factors to account for ADHD [50]. A priori, this explanation allows the blame to be placed on the brain, and at the same time absolves parents, and teachers, for the child’s disruptive behaviors [51]. But for illnesses such as adult psychosis, anti-stigma campaigns that present mental illness as an illness like any other have actually increased the perception of dangerousness and the desire to keep patients at arm’s length [52]. This paradoxical effect appears to be linked to stereotypes such as dangerousness associated with mental illness, which are more linked to biological than psychosocial explanations [53]. Although no studies have been conducted on the effects of anti-stigma campaigns for ADHD, and a fortiori in the context of Chinese culture, we will jointly develop with our Chinese partners a literacy program that is as balanced as possible in the causal explanations of ADHD and its treatments.

### Strength and limitations

We used CFIR as a theoretical framework to systematically capture the complexity of implementation of the project. We engaged the perspective of different clinicians (i.e., pediatricians, GPs, and nurses) from different level of hospitals to strengthen the trustworthiness of results and research uptake at the designated settings. The research team used the member check approach after writing the result section to ensure research participants confirm the outcome of the focus groups and therefore increase credibility of the findings. Coding was conducted by two researchers to enhance dependability of the findings of the study. Discrepancies identified between these coders were resolved in team meeting with other researchers.

Limitations of the study include the relatively small number of participants, which may reduce the range of perspectives. However, we recruited all stakeholders from the different sites. Above all, the small number of specialist physicians, general practitioners and nurses qualified in the field of ADHD dictated the number of participants. Because of their expertise, however, focus group participants were ideal for assessing the factors surrounding the barriers and facilitators to implementation of the ADHD Share Care Pathway program. The other limitation is the lack of direct involvement of patients and families. This study focused on stakeholders’ perceptions of the challenges and facilitators associated with implementing the intervention. It was the stakeholders who reported the “Patients’ Needs” in the “Outer Setting” domain. In general, CIFR does not make extensive use of patient perspectives or experience [32]. But the results of the study showed that the patient perspective will be essential in defining and evaluating mental health literacy programs, and the implementation of accommodation measures.

Due to the specific nature of the study context, the results are not transferable and generalizable to other care contexts, which would require specific projects. What is generalizable, however, is the application of CFIR in the specific perspective of a particular mental disorder (ADHD), in a distinctive medical setting (Developmental Behavioral Pediatric) and in a well-defined cultural and geographical context (Xinhua Hospital in Shanghai and behavioral pediatric services in Chongming and Ninghai).

How generalizable are the results of this study beyond the targeted hospitals? While China is a vast country, the Yangtze River Delta region, centered on Shanghai and including the provinces of Jiangus, Zheijang and Anhui, is about twice the size of South Korea, with a population of around 150 million. It is the region with the highest gross domestic product (GDP) in China (around that of Japan) and a per capita GDP comparable to that of Poland. The region’s integrated municipal system facilitates the expansion of healthcare models. For example, the Chongming branch of Xinhua Hospital (in Shanghai municipality) and Ninghai Maternity and Child Health Care Hospital (in Zhejiang province) are part of Xinhua Hospital’s medical consortia, providing regional medical services and ensuring the ongoing promotion and implementation of ADHD care programs. Another factor facilitating the spread of the model is the commitment to establishing collaborations between academic centers and community health service centers. For example, in January 2024, Jiao Tong University Medical School launched the first phase of a training network with all community centers in the Shanghai municipality. Finally, the fact that developmental behavioral pediatrics is a young specialty means that the model can be extended because it has no competition yet.

### Comparison between paediatric and psychiatric settings

In another article, we also explored the obstacles and facilitators to implementing the same model of ADHD care, but in a psychiatric setting in Beijing. Comparing these two settings, the first difference in implementation challenges is the varying level of qualification of physicians. Physician training varies between different levels of hospital, but not in the same way in psychiatric and pediatric settings. Some psychiatrists in tertiary hospitals have accumulated more experience, and patients are more likely to go to these hospitals, whereas physicians in secondary and primary psychiatric hospitals lack both experience and patients, creating imbalances in access to care. This contrast is even more pronounced in pediatrics. Pediatricians in secondary and primary hospitals have relatively little exposure to developmental and behavioral disorders, and ADHD in particular. In tertiary hospitals, resident pediatricians are generally required to rotate for a few months in the behavioral pediatrics department, but in secondary and primary hospitals, such training is relatively scarce. There are differences between provinces. In Shanghai, efforts are being made to rotate pediatricians working in level 1 and 2 hospitals into the pediatric wards of level 3 hospitals, so that they can receive training in developmental behavioral disorders. But as behavioral pediatrics is a very young specialty, even in comparison with pediatric psychiatry, these specialists represent only a tiny proportion of pediatricians.

The second difference is the lack of time and workload. In China, paediatric hospitals are often overcrowded, and doctors are used to rapid diagnosis and treatment decisions. In contrast, the medical process in psychiatric settings is relatively longer, with more elaborate discussions with patients and parents. As developmental psychiatry relies heavily on detailed assessment, combined with relatively lengthy developmental tests, taking the necessary time is essential for accurate diagnosis. Because they allocate different amounts of time per visit, and because their training differs, developmental psychiatrists and pediatricians do not see the same patients, even though they are both qualified to diagnose and treat ADHD. Developmental pediatricians focus on primary pediatrics, early childhood development, mental health promotion and certain disorders such as ADHD, autism and language development delays. Psychiatrists focus on complex cases of ADHD, associated with anxiety, depression and behavioral disorders, which developmental pediatricians generally tend to refer to them in tertiary centers. There is also an age difference in the children treated in the two systems. Developmental pediatricians deal mainly with pre-school and school-age children, while psychiatrists treat more school-age children and adolescents, as well as adults with ADHD.

Finally, we have also observed that obstacles linked to stigmatization and cultural differences in ADHD care are more readily raised by pediatricians than by psychiatrists. These differences may be explained in part by the fact that pediatricians practice in a more socially accepted medical context than psychiatrists. There is less public stigma attached to developmental paediatrics, with routine welfare visits for example, than to psychiatry. Contact with less stigmatized and isolated families may also explain why pediatricians were more readily able to identify certain Western approaches to ADHD care, such as accommodating or modifying expectations according to the child’s difficulties, as more likely not to be accepted in Chinese culture.

The implementation environment must therefore take account of different factors at each site. It is clear, however, that in China as in Canada, collaboration between specialists and general practitioners, as well as between pediatric specialists and psychiatrists, again following the same stepped care model, is essential for quality ADHD management. To this end, as in Canada [22–25], collaboration exists between the two disciplines. The annual meetings of the Chinese National Psychiatric Association and the Association of Developmental Behavioral Pediatrics provide a learning platform for mutual exchange between the two specialties. Each year, there are at least two national training sessions and several regional training sessions at provincial and municipal levels.

## Conclusions

This study provides findings of factors that facilitate and restrain the successful implementation of the Canadian ADHD Shared Care program in China. The key factors that contribute to the success of this program include strong leadership engagement, learning climate, cosmopolitanism, and alignment of the project’s goal with the knowledge users’ and governmental policies. The reported barriers can be used to inform knowledge users on how to improve implementation of the program. Key barriers include lack of physicians’ knowledge on ADHD principles, complexity of conduction of an international project, lack of public awareness and stigma toward ADHD, cultural differences between Canada and China, and lack of resources. Appropriate training of health care providers, adapting the program for the Chinese context, increasing public awareness about ADHD through social media platforms and schools as well as providing strong project management and guidelines that clearly describe the role and expectations of each team member are essential to successful implementation.

### Electronic supplementary material

Below is the link to the electronic supplementary material.

Supplementary Material 1

## Data Availability

The datasets used and/or analysed during the current study are available from the corresponding author on reasonable request.
