# Supplementary material for: Barriers and facilitators to implementing a Canadian shared-care ADHD program in pediatric settings in Shanghai: a consolidated framework for implementation research approach
Source: BMC Health Serv Res. 2024 May 2;24:564. doi: 10.1186/s12913-024-10910-7 (PMC11064246; doi:10.1186/s12913-024-10910-7)
Supplement: Supplementary file 1 — Supplementary Material 1 [file 12913_2024_10910_MOESM1_ESM.docx]

**Appendix A**

**CFIR Constructs**

**Characteristics of Individuals**

According to the CFIR, organizations are made up of individuals who are responsible for the implementation of an intervention, and thus, influence the success of the implementation. Within this Domain of the CFIR, we identified the constructs of Knowledge and Beliefs, Self-efficacy, and Other Personal Attributes, specifically, high stakeholder motivation.

Knowledge and Beliefs: This construct refers to individuals’ familiarity with the underlying principles of the intervention.

Self efficacy: Self-efficacy refers to individual’s belief in their own capability to complete course of action to achieve implementation goals.

Other personal attributes: This construct refers to broader personal attributes such as motivation, values, and learning style which can impact a successful implementation.

**Inner Setting**

This domain focuses on the dynamic interactions between the “working parts” within an organization which may influence implementation. The CFIR constructs relevant to the inner setting in our study were the Implementation Climate and Readiness for Implementation.

**Implementation Climate** : This construct is related to the absorptive capacity for change in engaged individuals, and the extent to which an organisation supports and rewards the use of a new intervention. Related to the CFIR’s Implementation Climate construct, we identified the sub-constructs of compatibility, organizational incentives, learning climate, and tension for change.

Compatibility: refers to the degree of fit between the value attributed to the intervention and stakeholders’ values and needs, as well as existing systems within the organisation.

Organisational incentives: are related to the extrinsic rewards for reinforcing behaviors in order to gain desirable results and consequently, success in implementation.

Learning climates: refers to leaders’ expression of their own fallibility and need for getting assistance and input from team members.

Tension for Change: relates to the degree to which stakeholders perceive that the current situation needs to be changed.

**Readiness for implementation:** This construct relates to indicators of organizational commitment to implement an intervention. We identified the sub-constructs of leadership engagement and available resources.

Leadership engagement: relates to commitment and involvement of leaders with the implementation.

Available resources: refer to the level of resources dedicated for implementation.

**Intervention Characteristics**

This domain pertains solely to the key attributes that make up an intervention. The CFIR constructs relevant to this domain in our study were the complexity of the intervention, the source of an intervention, and its relative advantage.

Complexity: This construct relates to the perceived difficulty of the implementation of this project.

Intervention source: Intervention source relates to the opinion of stakeholders about whether the intervention is externally or internally developed.

Relative advantages: This construct refers to stakeholders’ opinion on the benefits of implementing an intervention versus an altered or current solution.

**Outer Setting**

This domain refers to the outer setting’s network of support for implementation of an intervention including the social and political context. Pertain to this domain, we identified the constructs of cosmopolitanism, patient needs and resources, and external policies.

Cosmopolitanism: Within CFIR, cosmopolitanism is the degree to which an organization is networked with other external organizations.

Patients need and resources: This construct reflects patient-related factors of implementation of the intervention.

External polices: This construct encompasses external strategies to spread innovation.

Specific Barriers, Facilitators, and Mixed Factors that Contribute to the Implementation of a Shared Care Pathways Program in Shanghai

| CFIR Domain and Constructs | Facilitators | | Barriers | Mixed |
| --- | --- | --- | --- | --- |
| Characteristics of the Individual |  | |  |  |
|  |  | |  |  |
| -Knowledge and beliefs about the intervention |  | | - Lack of physicians’ knowledge in ADHD management. |  |
| - Self-efficacy |  | |  | - Confidence in their own capacity to execute courses of action to achieve the project’s goals. However, they need related support and training to do so. |
| - Other personal attributes | - Stakeholders’ high motivation to help their patients and contribute to their field and organization. - Roleplaying, online training and educational videos were identified as individuals’ preferred learning methods | |  |  |
| Inner Setting |  | |  |  |
| - Implementation Climate |  | |  |  |
| 1. *Compatibility* | - Physician values and aims were in alignment with project goals. | | The philosophy underling the treatment of ADHD children are different in Canada and China | Despite differences, physicians in China are willing to learn from the Canadian approach. |
| 1. *Organizational Incentives* | - The opportunity to participate in scientific research. - Increase salary and bonus. - Allocating more trained staff to help with assessment and screening for ADHD. - Receiving promotions. - Receiving certifications. | |  |  |
| 1. *Learning Climate* | - Potential leaders express their own fallibility and need for learning and collaboration with colleagues. | |  |  |
| 1. *Tension of change* | - strong urge for improving the situation. | |  |  |
|  |  | |  |  |
| -Readiness for Implementation |  | |  |  |
| 1. *Leadership engagement* | - Leaders are willing train others in the health system. - Leaders are willing to manage and coordinate an interdisciplinary team. | |  |  |
| 1. *Available Resources* | - Determining the level of understanding of GPs with regards to ADHD can facilitate providing appropriate resources. | | - Only a basic level of care is currently offered in community hospitals. - Heavy workload and insufficient specialized medical personnel. - Lack of qualified support staff. - Lack of training for general physicians. - Lack of standardized procedures to ADHD treatment. - Lack of availability of medication to treat ADHD in primary and secondary level hospitals. - Not havening licenses for certain ADHD medication. |  |
| Intervention Characteristics |  | |  |  |
| -Complexity |  | | - Time limitation for implementing the program. - Communication and cooperation between different teams in an international, multi-centered project. - Meeting the varying needs of different settings and hospitals. - Integration of resources at different levels of care. |  |
|  |  |  |  |  |
| -Intervention Source | - Stakeholders are willing to learn Canadian approach This | | - Program is externally developed (foreign); | - This program is externally developed (foreign); however, stakeholders are willing to learn Canadian approaches and implement it. |
| -Relative Advantage | - The introduction of a systematic approach to ADHD management. - Standardized treatment. - Increased training of physicians. - Increased participation of general physicians. | |  |  |
| Outer Setting |  | |  |  |
| -Cosmopolitanism | - The number of schools within the reach of hospital education networks is high in Shanghai and Chongming. - There exist good working relationships between physicians and schools in Shanghai and Chongming. - Implementation settings have a strong relationship with other levels of hospitals in the area. | | - Ninghai stakeholders do not have a strong relationship with schools. |  |
| -Patients’ needs and Resources |  | | - Lack of public awareness about ADHD. - Many individuals do not know that ADHD is treatable. - Metal health stigma. |  |
| -External Policies and Incentives | - Governmental policies are in place to increase overall training of GPs. | |  |  |
